# Adherence to denosumab therapy and all-cause mortality in dialysis patients with osteoporosis: a retrospective cohort exploratory study

**DOI:** 10.3389/fphar.2025.1708051

**Published:** 2026-01-08

**Authors:** Ying-Chou Chen, Jia-Feng Chen, Shan-Fu Yu, Chung-Yuan Hsu, Chien-Hua Chiu

**Affiliations:** 1 Department of Rheumatology, Kaohsiung Chang Gung Memorial Hospital, Kaohsiung Municipal Ta-Tung Hospital, Chang Gung University College of Medicine, Kaohsiung, Taiwan; 2 Department of Nephrology, Kaohsiung Chang Gung Memorial Hospital, Chang Gung University College of Medicine, Kaohsiung, Taiwan

**Keywords:** medication adherence, all-cause mortality, osteoporosis, dialysis, denosumab therapy

## Abstract

**Background:**

Dialysis patients have a high risk of osteoporosis, leading to increasedfracture and mortality rates. Denosumab is commonly used in thispopulation due to its lack of renal accumulation, but its long-termbenefit depends on sustained adherence. This This exploratory study investigated therelationship between denosumab adherence and all-cause mortality indialysis patients with osteoporosis.

**Methods:**

This retrospective case--control study included 1,200 hemodialysis patients. Four hundred deceased patientswere matched 1:3 by age, sex, and follow-up duration to 1,200 survivingcontrols. Adherence to denosumab therapy was calculated, and itsassociation with mortality was assessed using Cox regression analysis.

**Results:**

Among 401 denosumab-treated patients, 12 died and 389 survived duringfollow-up. The deceased group was older than survivors (85.0 ± 11.07 vs.75.87 ± 10.24 years, *p* = 0.016). Poor adherence occurred in 50% ofdeceased patients versus 7.5% of survivors (*p* <0.001).Comorbidities, including diabetes, hypertension, rheumatoid arthritis, liver disease, pulmonary disease, and cancer, differed significantlybetween groups. In Cox regression analysis, older age (HR = 1.137, 95%CI = 1.044–1.239) and poor adherence (HR = 0.065, 95% CI = 0.015–0.288) were significantly associated with higher mortality.

**Conclusion:**

Good adherence to denosumab therapy was associated with lower all-causemortality risk in dialysis patients withosteoporosis. Improving treatment adherence may enhance survival in this high-risk population. Given the exploratory nature of this study and the limited number of events, these findings require validation in larger cohorts.

## Graphical Abstract

**Figure:**
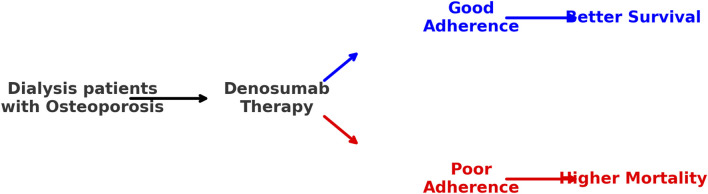
Schematic representation of the relationship between adherence to denosumab therapy and survival outcomes in dialysis patients with osteoporosis. Patients receiving denosumab were categorized into good adherence and poor adherence groups. Good adherence was associated with improved survival, whereas poor adherence was associated with increased mortality.

## Background

Osteoporosis is a major health concern that increases the risk of fractures and associated morbidity and mortality. It is also a frequent complication in patients undergoing hemodialysis (HD) (Walter et al., 2014). The incidence of fractures in HD patients has been reported to be higher than in the general population (Bolland et al., 2010a).

Denosumab, a monoclonal antibody targeting the receptor activator of nuclear factor kappa-B ligand (RANKL), is an effective therapy for osteoporosis in both postmenopausal women and men (Boyle et al., 2003; Kostenuik et al., 2009; Delmas, 2008; Bone et al., 2008; McClung et al., 2006). By binding to RANKL, denosumab prevents its interaction with the RANK receptor on osteoclasts, thereby reversibly inhibiting osteoclast-mediated bone resorption. While several anti-osteoporotic agents are available, most are contraindicated in patients with advanced chronic kidney disease due to drug accumulation.

Denosumab has demonstrated superior efficacy in increasing bone mineral density (BMD) and reducing fracture risk in the general population (Reid et al., 2010a; Sankaran et al., 2010; Bolland et al., 2010b). Importantly, impaired renal function has little effect on the pharmacokinetics of denosumab (Reid et al., 2010b), suggesting that its benefits in HD patients should be comparable to those in individuals with normal renal function (Bolland et al., 2010c; Wang et al., 2021).

Non-adherence to treatment can substantially diminish the therapeutic benefits of medication (Gordis, 1979; Haynes et al., 1980). In chronic diseases, non-adherence rates may reach 50% (Kripalani et al., 2007). Medication adherence is often quantified using the medication possession ratio (MPR), defined as the ratio of the number of doses dispensed to the total days in the dispensing period. Notably, previous research has shown that hip fracture risk increases by 0.4% for every 1% decrease in MPR (Rabenda et al., 2008). In the United States, poor compliance has also been linked to increased healthcare costs and hospitalization risk (Briesacher et al., 2007; Huybrechts et al., 2006; Sunyecz et al., 2008; Halpern et al., 2011).

Given these findings, this study aimed to investigate the association between adherence to denosumab therapy and all-cause mortality in patients with osteoporosis undergoing dialysis.

## Materials and methods

### Study design and population

This retrospective case--control exploratory study included patients with both dialysis and osteoporosis treated at Chang Gung Memorial Hospital between 2008 and 2016. The study was approved by the Chang Gung Memorial Hospital Institutional Review Board. A total of 1,200 patients patients on maintenance hemodialysis were identified from the nephrology department database and followed from the date of enrollment until death or the end of the observation period (December 2016), whichever occurred first.

The case group comprised 400 patients who died during follow-up. Each case was matched by age, sex, and follow-up duration to three surviving controls, yielding a 1:3 case--control design. Data collected included age, sex, body mass index (BMI), comorbidities (hypertension, diabetes mellitus, liver disease, pulmonary disease, rheumatoid arthritis, cardiovascular disease, and cancer), and denosumab use.

### Diagnostic criteria

Dialysis---Chronic kidney disease was defined according to established guidelines (Nelson and Tuttle, 2006), based on evidence of kidney damage and/or reduced kidney function (glomerular filtration rate GFRGFR), irrespective of the underlying cause.

Osteoporosis---Osteoporosis was diagnosed if the bone mineral density (BMD) T-score was ≤ −2.5 at the lumbar spine, femoral neck, or total hip, as measured by dual-energy X-ray absorptiometry (DEXA).

### Inclusion and exclusion criteria

#### Inclusion criteria

1. Diagnosis of osteoporosis.
2. Receiving maintenance dialysis hemodialysis.

#### Exclusion criteria

1. Active systemic infection.
2. History of cerebral hemorrhage or stroke.

#### Assessment of adherence

Adherence was evaluated according to the definition proposed by Cramer et al. (2008) incorporating both compliance and persistence:

- Compliance was measured using the medication possession ratio (MPR), calculated as the number of days the patient had medication available divided by the total number of days in the observation period. An annual MPR ≥80% was considered good compliance, whereas MPR <80% was defined as poor compliance. Prescription and refill data were obtained from the hospital’s electronic medical record and pharmacy dispensing system.
- Persistence was defined as the time from treatment initiation to discontinuation, without a medication refill gap of ≥30 days. The 30-day gap is a widely accepted threshold in adherence research, reflecting a clinically meaningful interruption in therapy (Cramer et al., 2008).

The 1-year MPR was calculated as the proportion of days with an available drug supply during the first 365 days of denosumab therapy. Persistence rate (PR) was defined as the proportion of patients who continued therapy without a ≥30-day gap. Risk factors for poor adherence (MPR <80%) were assessed at the end of year 1.

### Statistical analysis

All statistical analyses were performed using SPSS version 22.0 (IBM Corp., Armonk, NY, United States). Conditional logistic regression was used to adjust for potential confounders and evaluate the association between denosumab adherence and all-cause mortality. Statistical significance was set at *p* <0.05. Given the limited number of events (n = 12 deaths in the denosumab group), the multivariate Cox regression model, which included multiple covariates, should be interpreted with caution due to the risk of overfitting. The results are presented as exploratory and hypothesis-generating.

## Results

A total of 1,200 patients undergoing hemodialysis were identified, among whom 401 received denosumab therapy. At the end of follow-up, 12 patients in the denosumab group had died, while 389 remained alive. The mean age was significantly higher in the deceased group than in the survivors (85.0 ± 11.07 vs. 75.87 ± 10.24 years, *p* = 0.016). The proportion of females did not differ significantly between groups (75.0% vs. 81.2%, *p* = 0.405).

Overall, 35 patients demonstrated poor adherence to denosumab therapy. Poor adherence was markedly more common in the deceased group (50.0%) compared with the survivors (7.5%, *p* <0.001). Significant between-group differences were also observed in the prevalence of diabetes mellitus, hypertension, rheumatoid arthritis, liver disease, pulmonary disease, and cancer. Cardiovascular disease was more frequent in the deceased group (41.7% vs. 9.3%, *p* = 0.004) (Table 1).

**TABLE 1:** Clinical characteristics of study patients: deceased vs. surviving groups.

Variables	Deceased (n = 12)	Surviving (n = 389)	p-value
Age (years)	85.0 ± 11.07	75.87 ± 10.24	0.016
Gender (female, %)	9 (75.0)	316 (81.2)	0.405
BMI (kg/m^2^)	23.51 ± 3.34	23.97 ± 3.63	0.665
Adherence to therapy (poor, %)	6 (50.0)	29 (7.5)	<0.001
Smoking (%)	2 (16.7)	17 (4.4)	0.106
Alcohol consumption (%)	2 (16.7)	11 (2.8)	0.053
Rheumatoid arthritis (%)	2 (16.7)	27 (6.9)	0.213
Diabetes (%)	3 (25.0)	75 (19.3)	0.423
Hypertension (%)	5 (41.7)	176 (45.2)	0.523
Liver disease (%)	1 (8.3)	26 (6.7)	0.572
Cardiovascular disease (%)	5 (41.7)	36 (9.3)	0.004
Pulmonary disease (%)	1 (8.3)	32 (8.2)	0.649
Cancer (%)	2 (16.7)	40 (10.3)	0.364

**FIGURE 1:**
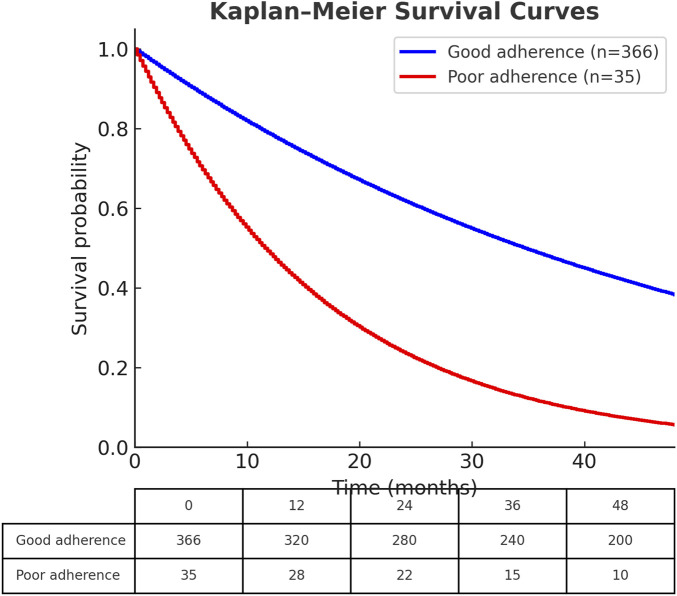
Kaplan--Meier survival curves comparing dialysis patients with osteoporosis who had good adherence (n = 366) versus poor adherence (n = 35) to denosumab therapy. Patients with good adherence demonstrated significantly better survival than those with poor adherence (log-rank test, *p* <0.001). The number of patients at risk is presented below the survival curves at 12-month intervals up to 48 months of follow-up. The small number of events in the poor adherence group (n = 6 deaths) necessitates cautious interpretation.

In Cox regression analysis, both older age (HR = 1.137; 95% CI = 1.044–1.239) and poor adherence (HR = 0.065; 95% CI = 0.015–0.288) were independently associated with increased mortality risk (Table 2). Kaplan--Meier survival curves demonstrated that patients with good adherence had significantly better survival compared with those with poor adherence (Figure 1).

**TABLE 2:** Multivariate cox regression analysis of mortality risk factors.

Variable	B	SE	Wald	p-value	HR (95% CI)
Adherence (poor)	−2.732	0.759	12.961	<0.001	0.065 (0.015–0.288)
Sex (female)	1.531	0.872	3.085	0.079	4.624 (0.837–25.528)
Age	0.129	0.044	8.595	0.003	1.137 (1.044–1.239)
BMI	−0.113	0.094	1.444	0.229	0.893 (0.743–1.074)
Smoking	0.299	1.145	0.068	0.794	1.349 (0.143–12.729)
Alcohol consumption	1.656	1.299	1.625	0.202	5.240 (0.411–66.873)
Diabetes	0.564	1.006	0.315	0.575	1.759 (0.245–12.642)
Hypertension	−0.990	0.761	1.693	0.193	0.372 (0.084–1.651)
Rheumatoid arthritis	1.499	1.091	1.887	0.170	4.475 (0.528–37.968)
Liver disease	1.207	1.156	1.091	0.296	3.345 (0.347–32.230)
Cancer	−0.900	1.160	0.602	0.438	0.407 (0.042–3.947)
Cardiovascular disease	1.391	0.704	3.911	0.048	4.020 (1.012–15.962)
Pulmonary disease	−0.672	1.162	0.335	0.563	0.511 (0.052–4.977)

HR, hazard ratio; SE, standard error; BMI, body mass index. Caution: The model includes 12 events and >8 variables; results should be interpreted as exploratory due to potential overfitting.

Causes of Death Among the 12 deceased patients, the primary causes of death were cardiovascular events (n = 5, 41.7%), infections (n = 3, 25.0%), cerebrovascular accidents (n = 2, 16.7%), cancer (n = 1, 8.3%), and unknown causes (n = 1, 8.3%).

## Discussion

This study evaluated the relationship between denosumab adherence and all-cause mortality in patients undergoing dialysis with concomitant osteoporosis. We found that high adherence to denosumab therapy was associated with a significantly lower mortality risk over a minimum 2-year follow-up. However, the small number of mortality events and the exploratory nature of the analysis necessitate caution in interpreting these findings.

Patients with CKD are at increased risk of osteoporosis and fractures (Goldenstein et al., 2015), but treatment decisions are complicated by CKD-related mineral and bone disorder. The KDIGO 2017 guidelines recommend individualized anti-osteoporosis treatment in patients with advanced CKD, ideally after a bone biopsy to avoid complications such as adynamic bone disease (Isakova et al., 2017). While denosumab’s long-term safety and efficacy have been well demonstrated in the general osteoporotic population (Broadwell et al., 2021), evidence in dialysis patients remains limited.

Adherence and persistence with antiresorptive therapy are critical for reducing fracture risk. Denosumab has a potential advantage in adherence due to its twice-yearly injection schedule, yet discontinuation is associated with rapid bone loss and increased fracture risk. It is important to note that for injectable medications like denosumab, treatment continuity is influenced by both patient behavior and systemic factors such as clinic scheduling and physician follow-up. Previous studies have linked good adherence to anti-osteoporosis medication with reduced mortality; however bisphosphonates (Morizio et al., 2018), evidence specific to denosumab in dialysis populations has been sparse. Our findings support that better adherence is associated with improved survival, possibly through fracture prevention and maintenance of functional status.

One plausible mechanism is the mitigation of fracture-related complications, which can worsen prognosis in dialysis patients. Additionally, denosumab may influence vascular calcification, as suggested by studies reporting regression of osseous and vascular calcifications in CKD patients with secondary hyperparathyroidism (Chen et al., 2020). The RANKL/RANK/OPG pathway is also expressed in vascular smooth muscle cells (VSMCs), and preclinical studies suggest that RANKL inhibition may modulate VSMC calcification. This could contribute to reduced cardiovascular mortality, which is a major cause of death in this population.

Our study has several limitations. First, as an observational study, causality cannot be established, and residual confounding (e.g., smoking, alcohol use, body mass index, duration of dialysis, frailty, nutritional status, and concomitant medications) is possible. Second, the small number of mortality events in the denosumab group and the inclusion of multiple covariates in the regression model increase the risk of overfitting and limit the statistical reliability of the hazard ratios. Third, reimbursement criteria under Taiwan’s NHI program limit our findings to severely osteoporotic patients, restricting generalizability. Fourth, biochemical bone turnover markers and bone biopsy data were unavailable, precluding evaluation of mineral metabolism changes related to CKD-MBD. Finally, bone mineral density and fracture outcomes were not analyzed, which limits mechanistic interpretation.

Despite these limitations, our findings highlight the importance of maintaining high adherence to denosumab therapy in dialysis patients with osteoporosis. Efforts such as patient education, integration of dental care, and follow-up reminders may help sustain persistence and improve long-term outcomes.

Conclusion In this exploratory retrospective case–control study, high adherence to denosumab therapy was independently associated with reduced all-cause mortality in dialysis patients with osteoporosis. Strategies to improve adherence should be prioritized to maximize treatment benefits in this vulnerable population. Future prospective studies with larger cohorts and longer follow-up are needed to confirm these observations.

## Data Availability

The original contributions presented in the study are included in the article/supplementary material, further inquiries can be directed to the corresponding authors.
